# Alternative Splicing of the Cardiac Sodium Channel Creates Multiple Variants of Mutant T1620K Channels

**DOI:** 10.1371/journal.pone.0019188

**Published:** 2011-04-28

**Authors:** Stefan Walzik, Annett Schroeter, Klaus Benndorf, Thomas Zimmer

**Affiliations:** Institute of Physiology II, University Hospital Jena, Friedrich Schiller University, Jena, Germany; Istituto Dermopatico dell'Immacolata, Italy

## Abstract

Alternative splicing creates several Na_v_1.5 transcripts in the mammalian myocardium and in various other tissues including brain, dorsal root ganglia, breast cancer cells as well as neuronal stem cell lines. In total nine Na_v_1.5 splice variants have been discovered. Four of them, namely Na_v_1.5a, Na_v_1.5c, Na_v_1.5d, and Na_v_1.5e, generate functional channels in heterologous expression systems. The significance of alternatively spliced transcripts for cardiac excitation, in particular their role in *SCN5A* channelopathies, is less well understood. In the present study, we systematically investigated electrophysiological properties of mutant T1620K channels in the background of all known functional Na_v_1.5 splice variants in HEK293 cells. This mutation has been previously associated with two distinct cardiac excitation disorders: with long QT syndrome type 3 (LQT3) and isolated cardiac conduction disease (CCD). When investigating the effect of the T1620K mutation, we noticed similar channel defects in the background of hNa_v_1.5, hNa_v_1.5a, and hNa_v_1.5c. In contrast, the hNa_v_1.5d background produced differential effects: In the mutant channel, some gain-of-function features did not emerge, whereas loss-of-function became more pronounced. In case of hNa_v_1.5e, the neonatal variant of hNa_v_1.5, both the splice variant itself as well as the corresponding mutant channel showed electrophysiological properties that were distinct from the wild-type and mutant reference channels, hNa_v_1.5 and T1620K, respectively. In conclusion, our data show that alternative splicing is a mechanism capable of generating a variety of functionally distinct wild-type and mutant hNa_v_1.5 channels. Thus, the cellular splicing machinery is a potential player affecting genotype-phenotype correlations in *SCN5A* channelopathies.

## Introduction

Voltage-gated sodium (Na^+^) channels mediate the fast upstroke of action potentials in electrically excitable cells by rapidly increasing the Na^+^ permeability [1]. The tetrodotoxin (TTX) resistant isoform Na_v_1.5, encoded by the *SCN5A* gene, is the predominant isoform in the heart [2], [3]. This channel plays a key role for excitability of atrial and ventricular cardiomyocytes and for rapid impulse propagation through the specific conduction system. Mutations in *SCN5A* can cause a broad variety of pathophysiological phenotypes, such as long QT syndrome type 3 (LQT3), Brugada syndrome (BrS), cardiac conduction disease (CCD), or sick sinus syndrome (SSS) [4], [5].

Heterologous expression of respective hNa_v_1.5 mutant channels and electrophysiological measurements often provided a reasonable explanation for the clinical phenotype. However, remarkable inconsistencies were noticed in several cases, like a severe disease manifestation in patients, but only subtle alterations in channel kinetics [6]–[8], or asymptomatic mutation carriers, but severely impaired in vitro channel function [9]–[12]. Even a broad spectrum of disease manifestations could be diagnosed in carriers of the same *SCN5A* mutation [11], [13]–[16]. Such apparent genotype-phenotype disassociations are most likely due to experimental limitations intrinsic to heterologous expression systems. In contrast to host cells like HEK293 or *Xenopus* oocytes, cardiomyocytes of the intact heart can be faced with additional risk factors or equipped with protective mechanisms capable of compensating for an impaired Na^+^ channel function, thereby aggravating the phenotype or preventing the onset of the disease in mutant gene carriers. Apart from known determinants such as gender or ageing [4], [10], risk factors relevant for disease manifestation and cardiac events could be: a) a deficient interaction with one of the cardiac proteins modulating hNa_v_1.5 [17], b) polymorphisms in hNa_v_1.5 [18], [19] or in other cardiac genes [6], c) a drop of intracellular pH [20], d) an increased temperature due to a feverish infection [21], e) drugs and xenobiotics [22], [23], f) the cellular phosphorylation status [24], [25], or g) a variable channel expression from the wild-type versus the mutated allele [26], [27].

Another important mechanism affecting genotype-phenotype correlations in *SCN5A* channelopathies could be the alternative splicing of wild-type and mutant Na_v_1.5 transcripts. To date totally nine Na_v_1.5 splice variants have been discovered in various tissues including heart, brain, dorsal root ganglia, breast cancer cells, and neuronal stem cells [28]. The abundance of an individual splice variant, relative to authentic Na_v_1.5, depends on the mammalian species, on the tissue, and on the ontogenetic stage. Although the physiological significance of alternative splicing of hNa_v_1.5 in the heart and in other tissues is less well understood, electrophysiological investigations revealed that functional consequences of Na_v_1.5 splicing are diverse: Spliced channels can show altered kinetics (Na_v_1.5a, Na_v_1.5d, Na_v_1.5e), they can be non-functional (Na_v_1.5b, Na_v_1.5f, truncated variants E28B-D), or their electrophysiological properties can be virtually unchanged (Na_v_1.5a, Na_v_1.5c), when compared to non-spliced Na_v_1.5 [28].

**Figure 1:**
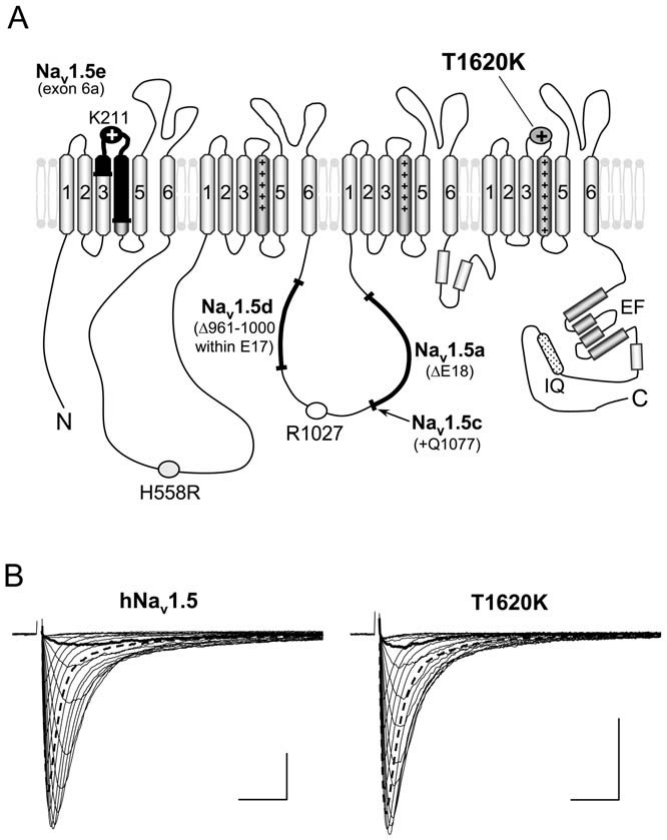
Proposed membrane topology of hNa_v_1.5 splice variants (A) and representative whole-cell currents through wild-type hNa_v_1.5 and mutant T1620K channels (B). (A) Four functional Na_v_1.5 splice variants have been detected so far: Alternative splicing can result in skipping of exon 18 (Na_v_1.5a), in alternative usage of the exon 18 splice acceptor site and the extension of this exon by a CAG trinucleotide coding for Q1077 (Na_v_1.5c), in partial deletion of exon 17 (Na_v_1.5d), and in the alternative usage of neonatal exon 6a instead of the adult exon 6b (Na_v_1.5e) [28]. Incorporation of neonatal exon 6a modifies the cardiac Na^+^ channel at 7 positions [32], [33]. Alterations in kinetics are largely due to the introduction of a positive lysine instead of an aspartate in the DI/S3S4 linker at position 211 [43]. Abbreviations: E17 - exon 17 sequence, E18 - exon 18 sequence, EF - EF-hand domain, IQ - calmodulin binding motif. (B) Representative Na^+^ currents generated by hNa_v_1.5 and mutant T1620K channels in HEK293 cells. T1620K channels inactivated faster at less depolarized potentials (thick line), but more slowly at more depolarized potentials (dashed line), when compared to hNa_v_1.5 (hNa_v_1.5: τ_h −50 mV_ = 4.35 ms, τ_h 0 mV_ = 0.80 ms; T1620K: τ_h −50 mV_ = 1.55 ms, τ_h 0 mV_ = 0.99 ms; see also Fig. 2B for the partial loss of the voltage dependency of open-state inactivation). Other parameters and statistics on both channel types are given in Fig. 2 and Table 2. Calibration bars indicate 2 ms and 1 nA.

In the present study we systematically investigated electrophysiological properties of a hNa_v_1.5 mutant (T1620K) in the background of all known functional Na_v_1.5 splice variants (Na_v_1.5a, Na_v_1.5c, Na_v_1.5d, Na_v_1.5e; Fig. 1). This mutation has been previously associated with two distinct cardiac arrhythmias: Mutant gene carriers presented with LQT3, an *SCN5A* channelopathy associated with enhanced Na^+^ channel activity, and with CCD, associated with loss-of-function in the cardiac Na^+^ channel [29]. We have chosen the T1620K mutation in the hope to observe differential effects on gain-of-function and loss-of-function properties of T1620K in the background of the distinct splice variants.

## Methods

### Expression plasmids

All Na^+^ channel variants used in this study are listed in Table 1. Our original human Na_v_1.5 cDNA (hH1; kindly provided by Dr. George, Vanderbilt University) codes for glutamine at position 1027 [30], which is considered as a rare Na_v_1.5 variation [31]. Therefore, we first introduced an arginine at this position. Second, we removed Q1077 from the resulting cDNA variant to obtain the most common hNa_v_1.5 sequence, previously designated as hH1c (AY148488) [31]. This cDNA was used as wild-type hNa_v_1.5 in the present study (Table 1). To obtain the T1620K mutant variants, we introduced lysine at position 1620, as previously described [29]. Patients of this family also presented with polymorphism H558R on the same allele (Fig. 1) [29]. Therefore, we introduced an arginine at this position in all T1620K cDNA variants, similarly as previously described [29]. The polymorphism H558R had no effect on the electrophysiological properties of hNa_v_1.5 and mutant T1620K channels [18], [29]. All sequence deletions (complete exon 18, partial exon 17, ΔQ1077), point mutations (positions 558, 1027, 1077, and 1620), as well as the modifications at 7 positions in adult exon 6b, leading to the corresponding neonatal exon 6a sequence [32], [33], were done by recombinant PCR using suitable mutagenesis primers and a thermostable DNA polymerase with proofreading activity (Pfu DNA polymerase, Promega, Madison, USA), according to previously described experimental procedures [29], [34], [35]. The correctness of PCR derived sequences was confirmed by DNA sequencing. All channel variants, listed in Table 1, were placed under the control of the SV40 promoter in expression plasmid pTSV40G, a derivative of pTracerSV40 (Invitrogen) [34]. Vector pTSV40G contains the coding region of the enhanced green fluorescent protein (EGFP; Clontech) in a separate expression cassette to allow for selection of transfected cells.

**Table 1:** Structure of the Na^+^ channel variants used in this study.

Channel variant	Exon composition			Amino acid at position			
	Exon 6 variant (205–234)	Exon 17 (930–1076)	Exon 18 (1078–1130)	558	1027	1077	1620
hNa_v_1.5	adult exon 6b	present	present	H	R	ΔQ	T
T1620K	adult exon 6b	present	present	R	R	ΔQ	K
hNa_v_1.5a	adult exon 6b	present	Δexon 18	H	R	ΔQ	T
T1620Ka	adult exon 6b	present	Δexon 18	R	R	ΔQ	K
hNa_v_1.5c	adult exon 6b	present	present	H	R	Q	T
T1620Kc	adult exon 6b	present	present	R	R	Q	K
hNa_v_1.5d	adult exon 6b	Δ961–1000	present	H	R	Q	T
T1620Kd	adult exon 6b	Δ961–1000	present	R	R	Q	K
hNa_v_1.5e	neonatal exon 6a	present	present	H	R	Q	T
T1620Ke	neonatal exon 6a	present	present	R	R	Q	K

Previously we used the hH1 clone [30] as the reference hNa_v_1.5 sequence to investigate the effect of the T1620K mutation on channel kinetics [29]. The corresponding protein sequence includes amino acids encoded by exon 6b as well as H558, Q1027, and Q1077. T1620K carriers and unaffected family members presented with R558 and H558, respectively. As shown previously, the polymorphism at this position did not differentially affect channel kinetics [29].

### Heterologous expression in HEK293 cells

Human embryonic kidney cell line (HEK293 cell line, ATCC number CRL-1573, supplied by the Centre for Applied Microbiology and Research, Salisbury, Wiltshire, UK) were cultured in MEM (Gibco BRL) supplemented with 10% fetal bovine serum, 2 mM glutamine, 100 U/ml penicillin, 100 µg/ml streptomycin, and 0.25 µg/ml amphotericin B. Cells were transfected by a standard calcium phosphate precipitation method using 1 to 2 µg plasmid DNA per transfection (60 mm cell culture dishes). After an incubation time of 24 h, the transfection mixture was removed, and the cells were seeded onto poly-L-lysine coated glass coverslips and cultured in fresh growth medium. Currents were investigated 24 to 48 hours after transfection.

### Patch-clamp measurements

Electrophysiological recordings were performed with the patch-clamp technique on the stage of an inverted microscope (Axiovert 100, Carl Zeiss Jena GmbH, Germany) using an Axopatch 200B amplifier (Axon-Instruments, Foster City, USA). The measurements were carried out at room temperature. The bath solution contained (mM): 140.0 NaCl, 1.8 CaCl_2_, 1.0 MgCl_2_, 10.0 glucose, 10.0 HEPES, pH 7.4 (CsOH). The pipette solution contained (mM): 10.0 NaCl, 130.0 CsCl, 10.0 EGTA, 10.0 HEPES, pH 7.3 (CsOH). Currents were elicited by test potentials from -120 mV to 40 mV in 5 or 10 mV increments at a pulsing frequency of 1.0 Hz. We used only cells that produced a peak current amplitude <5 nA. Steady-state activation (m_∞_) was evaluated by fitting the Boltzmann equation
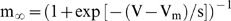
to the normalized conductance as function of voltage. Steady-state inactivation (h_∞_) was determined with a double-pulse protocol consisting of 500 ms prepulses to voltages between −120 and −30 mV followed by a constant test pulse of 10 ms duration to −20 mV at a pulsing frequency of 0.5 Hz. The amplitude of peak *I_Na_* during the test pulse was normalized to the maximum peak current and plotted as function of the prepulse potential. Data were fitted to the Boltzmann equation
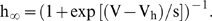
V is the test potential, V_m_ and V_h_ are the mid-activation and mid-inactivation potentials, respectively, and s the slope factor in mV. Glass pipettes were pulled from borosilicate glass and their tips were heat polished by the microforge MF 830 (Narishige, Japan). The pipette resistance was between 1.5 and 2.5 MΩ. Series resistance compensation was adjusted so that any oscillations were avoided leaving at most 25% of the series resistance uncompensated. Currents were on-line filtered with a cut-off frequency of 10 kHz (4-pole Bessel). Recording and analysis of the data was performed on a personal computer with the ISO2 software (MFK, Niedernhausen, Germany). The sampling rate was 50 kHz. Student's *t*-test was used to test for statistical significance. Statistical significance was assumed for P<0.05.

### Measurement of persistent Na^+^ currents

Determination of the persistent current fraction was done in *Xenopus laevis* oocytes using the two-microelectrode voltage clamp technique using a commercial amplifier (TEC-05-S; npi electronic GmbH, Tamm, Germany), as previously described [29], [36]. Briefly, *Xenopus laevis* oocytes were injected with undiluted cRNA preparations (∼1–2 µg/µl). The persistent current fraction was determined as follows: First, we measured the inward current at the end of a 200 ms test pulse that could be blocked by 10 µM TTX in 96 mM external Na^+^ (*I*_persistent_). Then, we reduced the extracellular Na^+^ concentration to 20 mM in order to insure adequate voltage control also for the first few milliseconds of the test pulse and determined the peak current amplitude in the same oocyte (*I*_transient_). Construction of the control plasmid encoding ΔKPQ channels was previously described [29].

## Results

**Figure 2:**
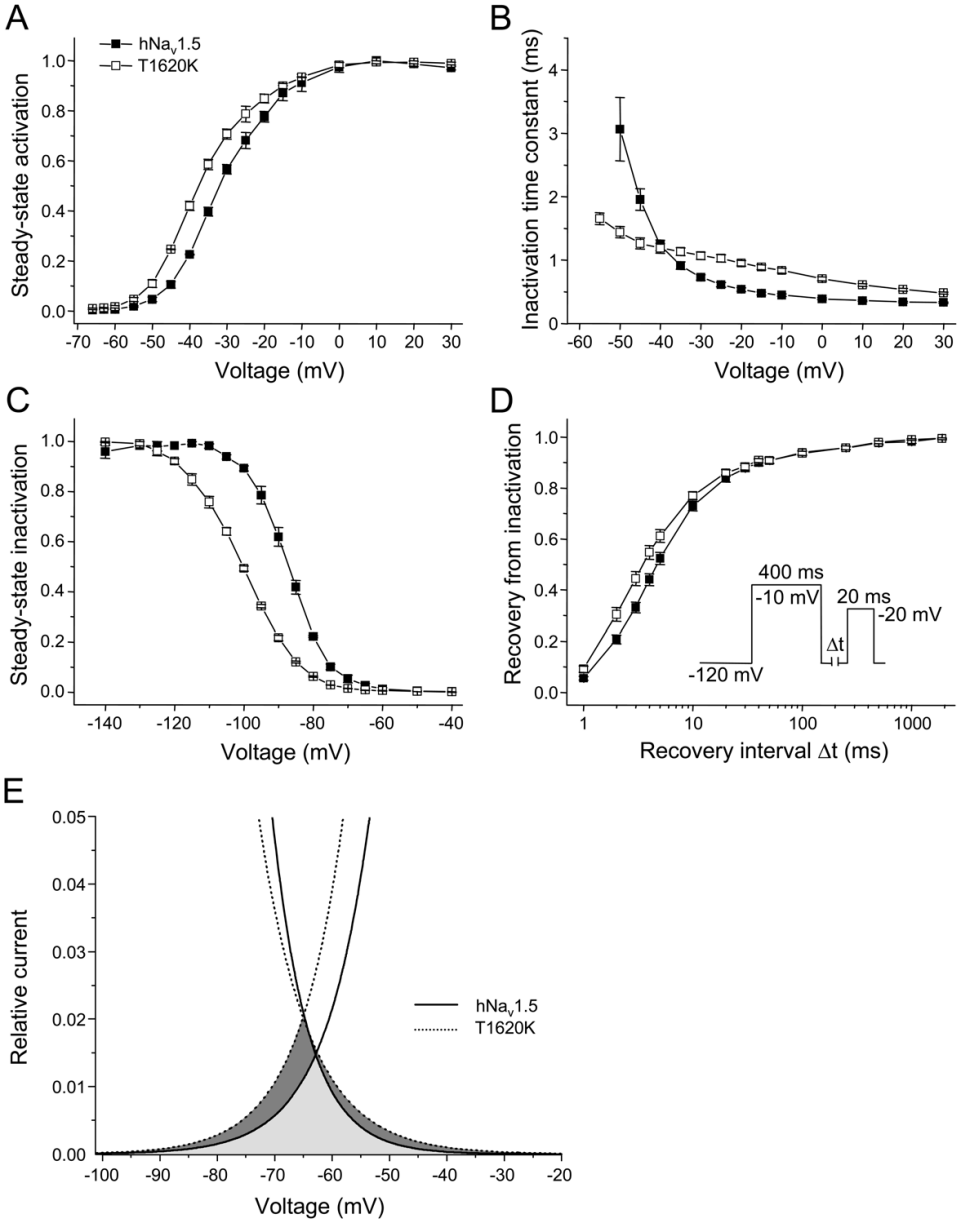
Electrophysiological properties of hNa_v_1.5 and mutant T1620K channels. (A) Steady-state activation as function of voltage. (B) Time constants of inactivation as function of voltage. The Na^+^ current decay was fitted with a monoexponential function. (C) Steady-state inactivation as function of voltage. (D) Recovery from inactivation. (E) Illustration of the window current in hNa_v_1.5 (light gray) and T1620K (dark gray). Due to the hyperpolarizing shift of steady-state activation (A) and the increased slope of the steady-state inactivation curve (C) both the amplitude and the voltage range of the window current is increased. Gain-of-function features, most likely resulting in prolonged ventricular action potentials and thus in LQT3, are the slower current decay at potentials positive to −40 mV (B), the faster recovery from inactivation (D), and the larger window current (E) [5], [29]. Loss-of-function features, most likely resulting in conduction slowing and thus in CCD, are the faster current decay at potentials negative to −40 mV (B), and the reduced steady-state availability at the resting membrane potential (C) [5], [29]. Individual curves illustrating steady-state activation (A), steady-state inactivation (C), and recovery from inactivation (D) were drawn using three representative measurements. Bars indicate S.E.M. For total number of measurements and statistical data evaluation see Table 2. The corresponding peak current densities are shown in Table 3.

First, we studied consequences of the T1620K mutation on electrophysiological properties of non-spliced wild-type hNa_v_1.5 in HEK293 cells by the whole-cell patch-clamp technique (Fig. 1B). We observed a negative shift of the mid-activation potential V_m_ and of the mid-inactivation potential V_h_ by −4.5 mV and −10.9 mV, respectively (Table 2; Fig. 2). The slope factor of the steady-state activation curve remained unchanged, whereas the steady-state inactivation curve flattened significantly. Recovery from inactivation was accelerated, which became apparent from the shorter time constant τ_f_. The most notable alteration was a substantial loss of the voltage-dependency of the inactivation time course (Fig. 2B), resulting in faster inactivation at hyperpolarized potentials (negative to −40 mV) and in slower inactivation at more depolarized potentials (positive to −40 mV). Peak current density was not affected by the mutation, and an increased persistent current fraction was not observed (Table 3). These data on T1620K are in close agreement with our previous measurements [29], when we used the original hH1 clone as the reference cDNA for heterologous expression [30]. The hH1 sequence corresponds to a very rare variation of hNa_v_1.5: It codes for Q1027, instead of the most commonly found R1027 variant [31]. Consequently, our data show that Q1027 and R1027 channels are indistinguishable with respect to the effect of the T1620K mutation.

**Table 2:** Electrophysiological properties of splice variants of hNa_v_1.5 and T1620K channels in HEK293 cells.

Channel	Steady-state activation			Steady-state inactivation			Recovery from inactivation				
	s (mV)	V_m_ (mV)	n	s (mV)	V_h_ (mV)	n	τ_f_ (ms)	A_f_	τ_s_ (ms)	A_s_	n
hNa_v_1.5	7.5±0.3	−31.4±1.3	14	6.0±0.1	−88.2±1.6	14	4.4±0.3	0.84±0.02	80.4±17.0	0.16±0.02	11
T1620K	7.5±0.3	−35.9±1.2#	11	8.3±0.2#	−99.1±1.7#	15	3.1±0.3#	0.84±0.01	73.2±21.3	0.16±0.01	11
hNa_v_1.5a	6.8±0.3	−33.2±1.0	13	6.2±0.2	−83.5±1.9	14	4.1±0.3	0.85±0.02	61.8±8.8	0.15±0.02	9
T1620Ka	7.1±0.2	−36.7±0.7#	32	8.3±0.2#	−99.3±1.5#	32	2.8±0.2#	0.84±0.01	71.3±14.2	0.16±0.01	15
hNa_v_1.5c	7.0±0.1	−32.8±0.3	33	6.2±0.1	−86.3±0.4	31	4.2±0.3	0.85±0.01	56.5±8.0	0.15±0.01	12
T1620Kc	7.7±0.2	−35.1±0.8#	36	8.1±0.1#	−00.3±1.3#	27	2.8±0.2#	0.83±0.01	68.6±8.6	0.17±0.01	20
hNa_v_1.5d	7.5±0.2	−23.5±0.7*	60	7.0±0.2*	−85.0±0.8	34	6.5±0.5*	0.75±0.10	121±46	0.25±0.10	5
T1620Kd	8.8±0.2§ #	−22.4±0.8§	55	9.2±0.3§ #	−99.8±1.5#	29	10.0±1.0§ #	0.77±0.01§	187±30§	0.23±0.02§	16
hNa_v_1.5e	8.3±0.4	−21.3±0.8*	8	6.0±0.2	−83.8±1.9	7	4.4±0.3	0.81±0.01	89.9±10.5	0.19±0.01	25
T1620Ke	8.8±0.5	−26.3±1.9§ #	8	8.1±0.5#	−100.9±1.2#	6	3.0±0.2#	0.82±0.02	91.4±21.0	0.18±0.02	10

*Indicates p<0.05 for a hNa_v_1.5 splice variant versus hNa_v_1.5, illustrating alterations caused by alternative splicing of hNa_v_1.5.

§ Indicates p<0.05 for a spliced T1620K variant versus T1620K, illustrating alterations caused by alternative splicing of T1620K.

# Indicates p<0.05 for a T1620K variant versus the corresponding hNa_v_1.5 variant, illustrating alterations caused by the T1620K mutation only.

**Table 3:** Peak current densities and persistent currents.

Channel	Peak current densities (HEK293)		*I*_persistent_/*I*_transient_ (×10^−3^)		
	pA/pF	n	at −30 mV	at −10 mV	n
hNa_v_1.5	271±25	29	11±1.9	21±6.3	6
T1620K	254±26	25	17±2.9	26±5.3	9
hNa_v_1.5a	347±48	16	8±1.4	17±5.1	7
T1620Ka	282±42	11	13±1.4	20±3.0	5
hNa_v_1.5c	295±28	35	15±6.6	20±7.3	11
T1620Kc	236±18	38	21±5.9	33±11	6
hNa_v_1.5d	110±15*	33	n.d.	n.d.	-
T1620Kd	84±11§	29	n.d.	n.d.	-
hNa_v_1.5e	194±20*	11	23±10	16±2.1	5
T1620Ke	182±23§	10	9±1.4	13±2.4	9

For measurements of persistent Na^+^ currents, we used the *Xenopus* oocyte system and undiluted cRNA preparations (∼1–2 µg/µl) for injection in order to increase this small current fraction. This resulted in transient Na^+^ currents >10 µA in 96 mM external Na^+^. In none of the channels investigated, a significant increase of the persistent current fraction could be observed. Low expression levels of hNa_v_1.5d and T1620Kd did not allow for an accurate determination of the persistent current fraction. As a positive control, we used cRNA for ΔKPQ channels, the first known LQT3 mutant. Respective values for *I*_persistent_/*I*_transient_ were: 179±42×10^−3^ at −30 mV, and 159±27×10^−3^ at −10 mV (n = 7).

*Indicates p<0.05 for a hNa_v_1.5 splice variant versus hNa_v_1.5.

§ Indicates p<0.05 for a spliced T1620K variant versus T1620K.

# Indicates p<0.05 for a T1620K variant versus the corresponding hNa_v_1.5 variant.

Beside Q1027, the hH1 sequence encodes also the amino acid Q1077. The additional CAG triplet is introduced by alternative usage of the exon 18 splice acceptor site [31]. The CAG-inclusive variant is considered as the splice variant Na_v_1.5c, because transcript levels of the CAG-exclusive variant predominate in the heart [31]. Electrophysiological properties of hNa_v_1.5 and hNa_v_1.5c were virtually identical (Table 2). Moreover, the effect of the T1620K mutation was independent of whether Q1077 was absent (T1620K) or present (T1620Kc; Table 2). In both T1620K and T1620Kc, the mid-activation and mid-inactivation potentials were shifted towards hyperpolarized potentials, the slope factor of the steady-state inactivation curve was increased, recovery from inactivation was accelerated, and channel inactivation was less voltage-dependent, when compared to hNa_v_1.5 and hNa_v_1.5c (Table 2). The persistent current fraction was not increased (Table 3).

**Figure 3:**
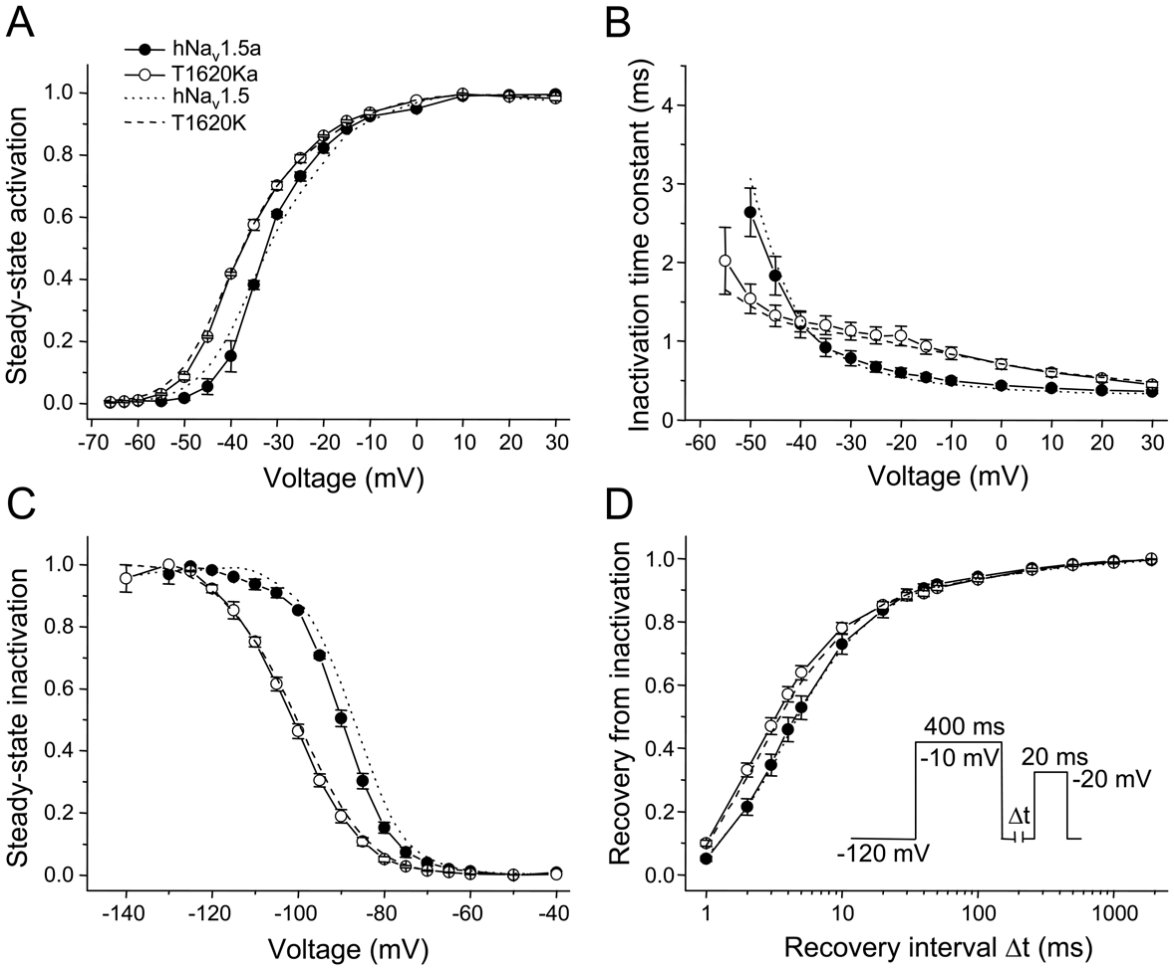
Electrophysiological properties of spliced hNa_v_1.5a and T1620Ka channels. Steady-state activation (A), time constants of inactivation (B), steady-state inactivation (C) and recovery from inactivation (D) were indistinguishable in hNa_v_1.5a and hNa_v_1.5 (dotted). Mutation T1620K produced similar defects in both the spliced and non-spliced (dashed) background (see Tables 2 and 3 for data evaluation and statistics).

Next we constructed splice variant hNa_v_1.5a and the corresponding mutant T1620Ka by deleting the exon 18 sequence in the hNa_v_1.5 and T1620K cDNAs, respectively. Similarly as found for hNa_v_1.5c, spliced Na_v_1.5a channels were indistinguishable from hNa_v_1.5. The introduction of mutation T1620K in the hNa_v_1.5a background, resulting in T1620Ka channels, produced similar electrophysiological defects as observed in T1620K channels (Fig. 3, Table 2). Together these data with T1620Ka and T1620Kc suggested that the positively charged lysine at position 1620 has a dominant effect on channel gating, independently of whether or not the intracellularly located DII-DIII linker is modified by alternative splicing (Fig. 1A).

**Figure 4:**
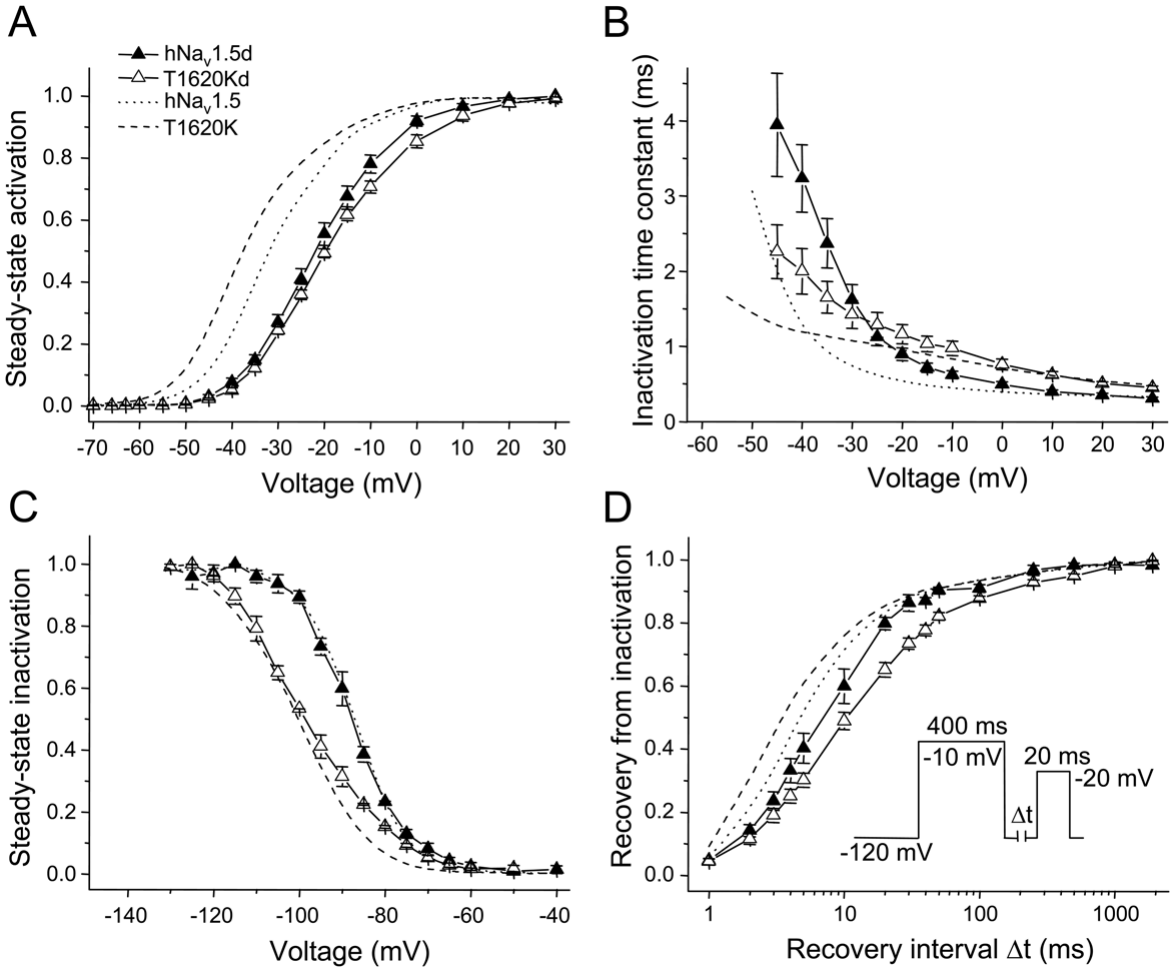
Electrophysiological properties of spliced hNa_v_1.5d and T1620Kd channels. Steady-state activation (A), time constants of inactivation (B), steady-state inactivation (C) and recovery from inactivation (D). In hNa_v_1.5d, the mid-activation potential was shifted towards depolarized potentials, and it remained unchanged in T1620Kd (A). Recovery from inactivation was slower in hNa_v_1.5d compared to hNa_v_1.5; it was further decelerated in T1620Kd (D). Note that the current densities were significantly reduced (Table 3), an effect that was largely due to a reduction in open probability in hNa_v_1.5d [34].

**Figure 5:**
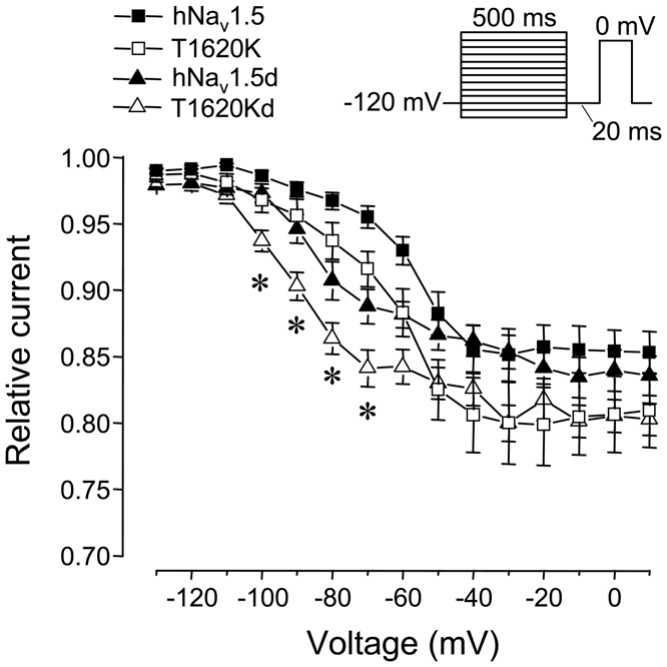
Steady-state slow inactivation. We first applied test pulses of 500 ms to achieve entry into the slow-inactivated state, followed by a short interval (20 ms) at −120 mV to allow only recovery from fast inactivation. Currents were recorded using a second test pulse to 0 mV and plotted against the potential of the first test pulse. Asterisks indicate significantly enhanced entry into the slow-inactivated state in T1620Kd channels *versus* hNa_v_1.5d, hNa_v_1.5, and T1620K channels. Bars indicate S.E.M.

However, when analyzing the effect of the T1620K mutation in the background of hNa_v_1.5d, a splice variant characterized by the deletion of a short stretch within the DII-DIII linker (Fig. 1) [34], we observed unexpected modulatory effects in the mutant splice variant. In hNa_v_1.5d, the deletion itself caused a positive shift of steady-state activation by 8.9 mV, and a significantly decelerated recovery from inactivation, when compared to full-length hNa_v_1.5 channels (Table 2) [34]. In the corresponding mutant splice variant T1620Kd, steady-state activation was not shifted towards hyperpolarized potentials and the slope factor of this curve increased significantly (Table 2, Fig. 4). Furthermore, in contrast to the accelerating effect of the T1620K mutation on recovery from inactivation in the other splice variants, the recovery time constant τ_f_ was increased in T1620Kd, when compared to splice variant hNa_v_1.5d (Table 2, Fig. 4). When compared to T1620K, also the recovery from slow inactivation was decelerated in T1620Kd, which became apparent from the larger time constant τ_s_ and the increased amplitude A_s_ (Table 2). Consistent with enhanced entry into the slow-inactivated state, we found most negative V_1/2_ values for steady-state slow inactivation in T1620Kd (−84.3±3.1 mV), compared to hNa_v_1.5d (−76.1±5.7 mV), T1620K (−69.1±4.2 mV), and hNa_v_1.5 (−60.0±2.0 mV) (Fig. 5). At membrane potentials between −100 mV and −70 mV, a significantly larger fraction of T1620Kd channels underwent slow inactivation, when compared to the other three types of channels (asterisks in Fig. 5). This indicates that alternative splicing of the mutant channel variant creates an additional loss-of-function feature that could be of physiological relevance at the resting membrane potential. In conclusion, effects of the T1620K mutation in the background of hNa_v_1.5d were different from those observed with mutated hNa_v_1.5, hNa_v_1.5a, and hNa_v_1.5c channels.

**Figure 6:**
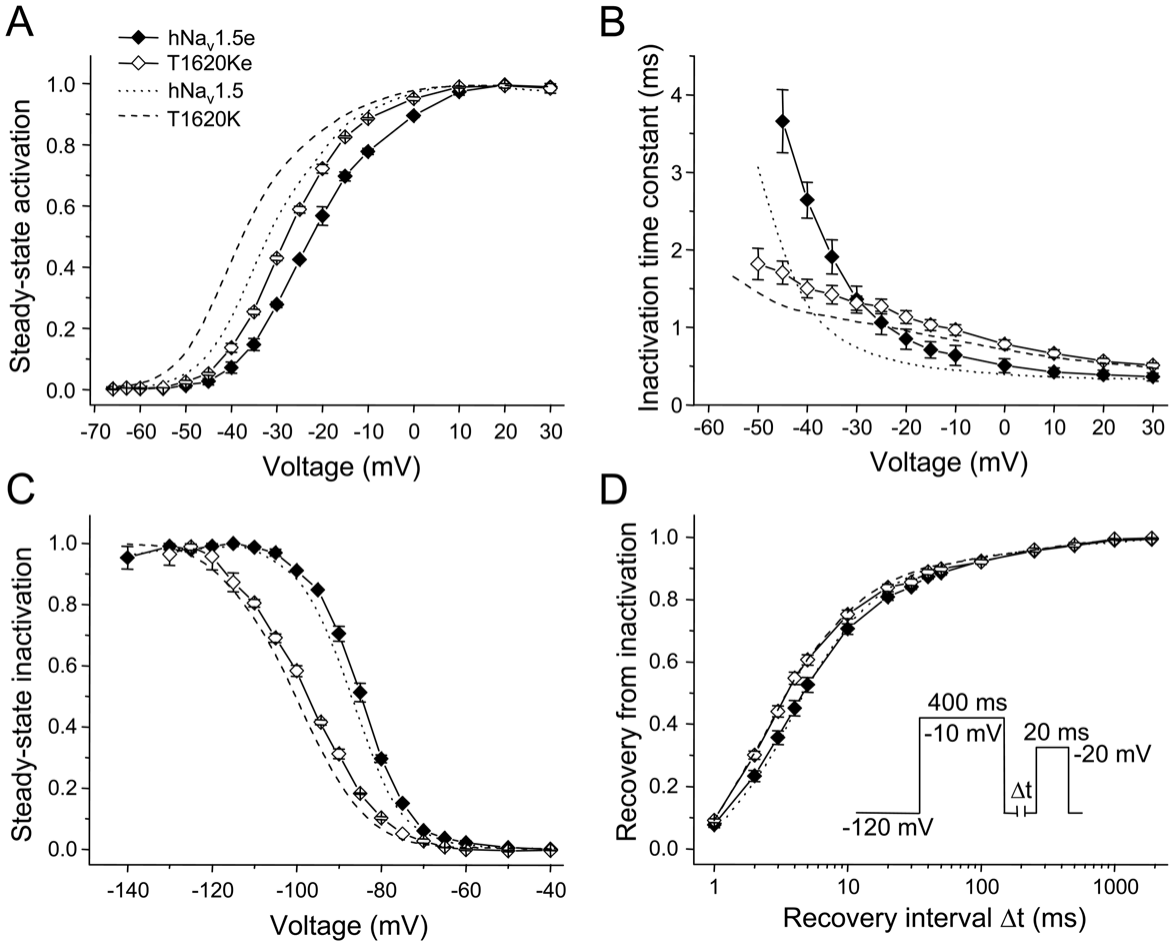
Electrophysiological properties of spliced hNa_v_1.5e and T1620Ke channels. Steady-state activation (A), time constants of inactivation (B), steady-state inactivation (C) and recovery from inactivation (D). In the neonatal variant hNa_v_1.5e, the mid-activation potential was shifted towards depolarized potentials, similarly as found for splice variant hNa_v_1.5d. Mutation T1620K caused a hyperpolarized shift of the steady-state activation.

In splice variant hNa_v_1.5e, the first of two tandemly arranged exon 6 sequences, neonatal exon 6a, is incorporated into the final transcript (Fig. 1A). Electrophysiological properties of hNa_v_1.5e channels were different from those of hNa_v_1.5 (Table 2, Fig. 6). We observed a pronounced shift of steady-state activation towards depolarized potentials by 10.1 mV in hNa_v_1.5e, and reduced peak current densities (Tables 2 and 3). Thus, electrophysiological properties of splice variant hNa_v_1.5e were similar to those of hNa_v_1.5d. However, unlike T1620Kd, the mutation at position 1620 caused a hyperpolarizing shift of steady-state activation and a faster recovery from inactivation in T1620Ke (Fig. 6A). Thus, T1620Ke channels show steady-state activation properties that are distinct from all other channel types investigated in this study. Steady-state inactivation and recovery from inactivation were similarly affected in T1620Ke, when compared to T1620K (Fig. 6C and D), and there was no increase of the persistent current (Table 3).

**Figure 7:**
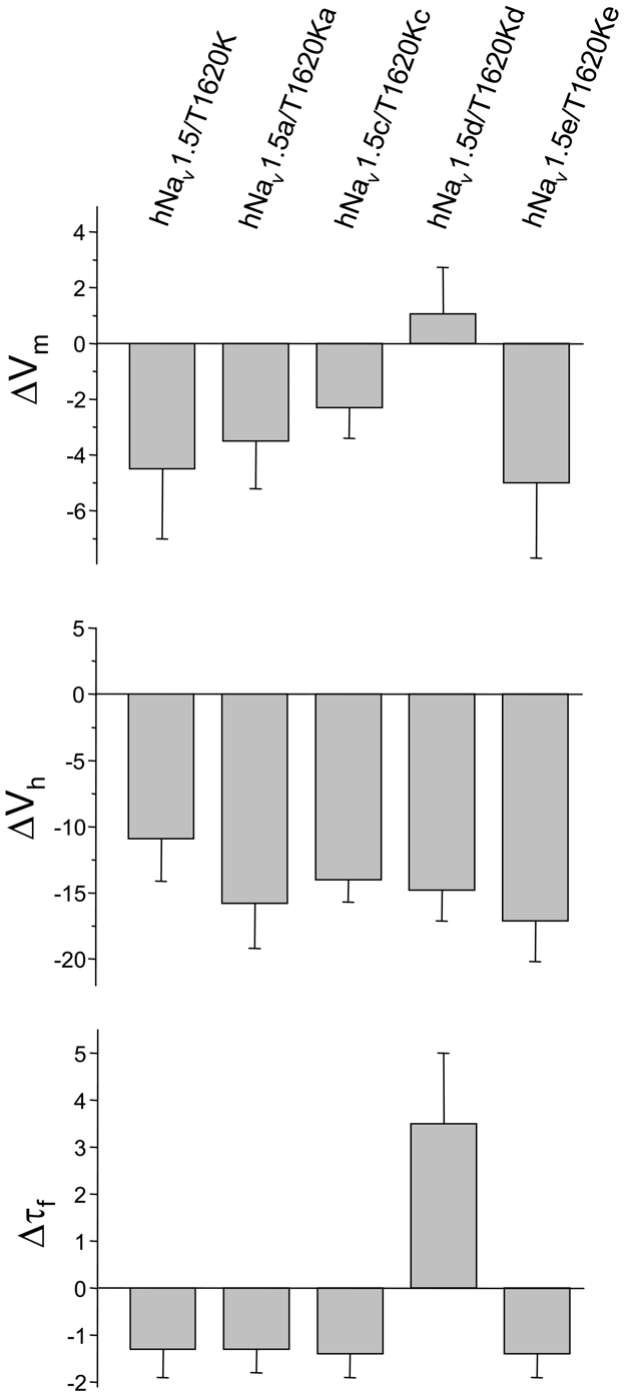
Relative alterations of mid-activation potentials V_m_, mid-inactivation potentials V_h_, and recovery time constants τ_f_. Mutation T1620K caused similar shifts in the background of most splice variants, except for hNa_v_1.5d. Bars indicate S.E.M.

In conclusion, alternative splicing of hNa_v_1.5 creates different wild-type and mutant channels. Statistical evaluation revealed a rather broad channel variability in case of the steady-state activation parameters and inactivation time constants (Table 2). Steady-state inactivation data were similar among all hNa_v_1.5 variants as well as among all mutated channels. Recovery from inactivation was accelerated in most of the mutant channels, except for T1620Kd channels (Fig. 4D). When considering only the relative alterations of the mid-activation potential V_m_, the mid-inactivation potential V_h_ and the recovery time constant τ_f_, mutation T1620K produced similar effects in the background of most splice variants (Fig. 7). In T1620Kd, however, V_m_ remained unchanged, and even an opposite effect on the recovery from inactivation was observed.

## Discussion

In the present study we systematically investigated electrophysiological properties of mutant T1620K channels in the background of all known functional Na_v_1.5 splice variants. We found that alternative splicing is a potential mechanism that allows generating a variety of functionally distinct wild-type and mutant hNa_v_1.5 channels.

The physiological significance of alternative splicing of hNa_v_1.5 in the heart and in other tissues is currently not fully understood [28]. Consequently, and this is a major limitation of the present study, we can only speculate on the function of alternative splicing in *SCN5A* channelopathies, in particular on the role of the functional T1620K splice variants for cardiac excitability in our patients. Recently, it became evident that alternative splicing of Na_v_1.5 is strictly species-dependent [2]. For example, Na_v_1.5a was not detected in the adult non-diseased human heart so far. Furthermore, there is still little information on the expression level and function of all five functional hNa_v_1.5 variants in different heart regions, at distinct ontogenetic stages, and in the diseased myocardium. Moreover, most of these splice variants were detected by RT-PCR only, but so far not by independent RNA or specific protein detection methods, like RNase protection assays or Western blotting, respectively. Except for Na_v_1.5e [37], specific antibodies do not exist. Shorter RT-PCR products can also result from intramolecular template switching by reverse transcriptases during cDNA preparation [38], [39]. Such PCR products were frequently misinterpreted in the literature as alternative transcripts [38], [39]. The pronounced tissue-, age- and species-dependent occurrence of shorter hNa_v_1.5 cDNA variants suggest that they are indeed the result of alternative splicing and that the proteins are produced in vivo, but an artifactual RT-PCR “splicing” cannot be fully excluded [28].

Na_v_1.5a is thought to be one of the major alternatively spliced Na_v_1.5 variants in the heart of small rodents [2], [28]. It has been also observed in several other tissues of rats and mice, such as dorsal root ganglia, brain or neuronal progenitor cell lines. Electrophysiological properties of hNa_v_1.5a were indistinguishable from those of authentic hNa_v_1.5, and gating of both channels was similarly affected by the threonine-lysine exchange at position 1620 (Figs. 3 and 7). These data suggest that, if this splice variant is produced in the human heart at all, it would not be of pathophysiological relevance for T1620K carriers.

Similar data were obtained for hNa_v_1.5c, the major Na_v_1.5 splice variant in the human heart [31], and for the corresponding splice variant T1620Kc. Noteworthy, the effect of Q1077 on electrophysiological properties of mutant channels has been already investigated by other groups and, depending on the background sequence, differential effects were observed [19], [20]. For example, three missense variants of hNa_v_1.5, identified in a SIDS cohort (delAL586-587, R680H, and V1951L), generated an increased persistent current only in the hNa_v_1.5 background (delQ1077), but not in the Q1077-inclusive variant hNa_v_1.5c [20]. These data suggest that deletion of Q1077 contributes to a disturbed open-state inactivation in mutant channels, as long as intracellularly located positions are affected. Position 1620 is located in an extracellular linker region and the fraction of the persistent current was neither increased in the hNa_v_1.5 nor in hNa_v_1.5c background (Table 3).

Na_v_1.5d is virtually absent in the heart of mice, rats, dogs, and pigs [2]. This variant was so far detected only in RNA preparations from the human heart, where it accounts for less than 3% of the total cardiac Na^+^ channel transcript pool [2], [28], [34]. Mutation T1620K resulted in unexpected effects in the hNa_v_1.5d background. Our data suggested that the short amino acid stretch, encoded by exon 17, is crucial for the function of the extracellular DIV/S3S4 linker. In contrast to the other mutant variants, mid-activation potential remained unchanged, recovery from inactivation was decelerated and steady-state slow inactivation was facilitated (Fig. 7). The latter effect was specific for voltages around the resting membrane potential (Fig. 5). These changes in kinetics in T1620Kd compared to T1620K would minimize three potential gain-of-function mechanisms expected to play a crucial role in LQT3: First, the window current in T1620Kd should be nearly absent. Respective steady-state activation and inactivation parameters suggest that the overlap between both curves is even less pronounced than that found for wild-type hNa_v_1.5 (Table 2). Second, recovery from inactivation was accelerated in T1620K and in many other LQT3 mutant channels [5], leading to facilitated channel reopening during the repolarization phase of the cardiac action potential [40]. In case of T1620Kd, such an effect can be excluded, because recovery from inactivation was even decelerated. And third, peak current densities were significantly smaller. A lower expression would counteract any of the potential gain-of-function mechanisms in LQT3. Consequently, T1620Kd channels are unlikely to contribute significantly to prolonged QTc values. Actually, mutant T1620Kd channels show most pronounced loss-of-function among all T1620K variants investigated in this study. Thus, cardiac conduction should be slower. In particular, the facilitated entry into the slow inactivated state and reduced current densities are features frequently found in hNa_v_1.5 mutant channels leading to CCD. In summary, it can be stated that, if this splice variant is expressed in the heart, T1620Kd channels would counteract QTc prolongation, but would also worsen cardiac conduction. The latter effect would be particularly important, if hNa_v_1.5d is specifically expressed in the cardiac conduction system, which was previously hypothesised [34].

Na_v_1.5e, also called neonatal Na_v_1.5 or nNa_v_1.5, is strongly expressed in neonatal mouse heart, but significantly down-regulated during development [37]. Our data with hNa_v_1.5e and T1620Ke suggest that the switch from neonatal to adult exon 6 results in an improved cardiac excitability. In the adult variants, hNa_v_1.5 and T1620K, peak current densities increased and steady-state activation was shifted towards hyperpolarized potentials. The latter effect is likely associated with an increased window current, contributing to QTc prolongation. Although the expression of Na_v_1.5e in the neonatal human heart has not yet been confirmed, it is interesting to speculate that *SCN5A* mutations, associated with sudden infant death syndrome (SIDS), may provoke more severe channel defects when the neonatal exon 6 is replaced by the adult variant after birth. Furthermore, clinically relevant mutations may be even introduced or replaced by a non-mutated sequence when one of the exon 6 variants itself is affected. Moreover, it is interesting to note that Na_v_1.5e is expressed in human neuroblastoma [33] and in highly metastatic human breast cancer cell lines [32]. Na_v_1.5e was also up-regulated in human breast cancer biopsy tissues, and a strong correlation was found between Na_v_1.5e expression and clinically assessed lymph node metastasis [32]. Assuming that hNa_v_1.5e is essential for the enhancement of migration and invasion [32], [41], [42], *SCN5A* loss-of-function mutations should exert protective effects against some types of cancer.

In conclusion, alternative splicing generates a variety of functionally distinct wild-type hNa_v_1.5 and mutant T1620K channels. The cellular splicing machinery should be considered as a potential player affecting genotype-phenotype correlations. At the same time we have to notice that conclusions with respect to the physiological significance from our results for this distinct *SCN5A* channelopathy are still restricted by our yet limited knowledge on cell-specific and age-dependent generation of splice variants in the normal and diseased human myocardium.
